# FirmiData: a set of 40 genomes of Firmicutes with a curated annotation of ICEs and IMEs

**DOI:** 10.1186/s13104-022-06036-w

**Published:** 2022-05-10

**Authors:** Gérard Guédon, Julie Lao, Sophie Payot, Thomas Lacroix, Hélène Chiapello, Nathalie Leblond-Bourget

**Affiliations:** 1 DynAMic, Université de Lorraine, INRAE, 54000 Nancy, France; 2 MaIAGE, Université Paris-Saclay, INRAE, 78350 Jouy-en-Josas, France

**Keywords:** Integrative and conjugative element, Integrative and mobilizable element, Mobile element, Conjugation

## Abstract

**Objectives:**

‘Integrative and Conjugative Elements’ (ICEs) and ‘Integrative and Mobilizable Elements’ (IMEs) are two classes of mobile genetic elements that are complex to detect and delineate. Therefore, they are yet poorly annotated in bacterial genomes. FirmiData provides to the scientific community of microbiologists and bioinformaticians a reference resource of annotated ICEs and of IMEs from Firmicutes. It illustrates their prevalence and their diversity but also gives information on their organization.

FirmiData was designed to assist the scientific community in identifying and annotating these elements by using the sequences of these ICEs and IMEs for the identification of related elements in other genomes of Firmicutes. Therefore, Firmidata meets the needs of the scientific community.

**Data description:**

Firmidata provides a manually curated annotation of 98 ICEs and 148 IMEs identified in 40 chromosomes of Firmicutes. The delineation at the nucleotide level of almost all of these elements allows for the characterization of the genes they carry.

## Objective

Unlike conjugative or mobilizable plasmids, ICEs and IMEs are difficult to detect because of their integration into replicons. This is why they have been neglected and even completely ignored for a long time.

However, efforts were made in the last 10 years to develop semi-manual [1, 2] and automatic [3–5] methods to search for conjugative elements in bacterial genomes. These led to the identification of an increasing number of ICEs and IMEs and to the demonstration of their diversity. We currently know that these elements are highly widespread in bacteria and archaea [6–8].

Despite these recent efforts, ICE and IME annotation is very rarely performed and released in public genome databases. The poor annotation of ICEs and IMEs within bacterial genomes is multifactorial. It results both from the extreme variability of their gene content and from the low specificity of their signature proteins. It is also due to their complex organization since they often form composite elements, with ICEs/IMEs nested within each other and/or organized in tandems.

The objective of FirmiData is to offer to both the communities of microbiologists and bioinformaticians a semi-manual annotation of a large dataset of ICEs and IMEs from Firmicutes. In this work, an expert analysis was done to verify, to characterize and to delimit all ICEs and IMEs they carry. The chromosomes of diverse groups of Firmicutes were chosen to illustrate the diversity of the elements as well as of their various organization into composite elements. The sequences of these elements can be used as reference for the identification of related elements in other genomes of Firmicutes*.*

## Data description

FirmiData is a set of 40 public annotated genomes of Firmicutes (information on strains are available in Table 1, 'Data file 41’, [9]). These genomes were extracted from the Refseq database for which ICEs and IMEs annotation was added using the standard annotation features and qualifiers used in the Genbank format [9].

**Table 1:** Data sets

Label	Name of data file	File types	Data repository and identifier
Data file 1	Cdi630.gb	GenBank (gb)	Data INRAE, https://doi.org/10.15454/VI7YRB [9]
Data file 2	CdiR20291.gb	GenBank (gb)	Data INRAE, https://doi.org/10.15454/VI7YRB [9]
Data file 3	DfoDMC.gb	GenBank (gb)	Data INRAE, https://doi.org/10.15454/VI7YRB [9]
Data file 4	EfalV583.gb	GenBank (gb)	Data INRAE, https://doi.org/10.15454/VI7YRB [9]
Data file 5	EfmISMMSVRE1.gb	GenBank (gb)	Data INRAE, https://doi.org/10.15454/VI7YRB [9]
Data file 6	FprA2-165.gb	GenBank (gb)	Data INRAE, https://doi.org/10.15454/VI7YRB [9]
Data file 7	Lca919.gb	GenBank (gb)	Data INRAE, https://doi.org/10.15454/VI7YRB [9]
Data file 8	LlaIO-1.gb	GenBank (gb)	Data INRAE, https://doi.org/10.15454/VI7YRB [9]
Data file 9	LmoLCC2378.gb	GenBank (gb)	Data INRAE, https://doi.org/10.15454/VI7YRB [9]
Data file 10	Lph3177T.gb	GenBank (gb)	Data INRAE, https://doi.org/10.15454/VI7YRB [9]
Data file 11	LspYL32.gb	GenBank (gb)	Data INRAE, https://doi.org/10.15454/VI7YRB [9]
Data file 12	RhoA2-183.gb	GenBank (gb)	Data INRAE, https://doi.org/10.15454/VI7YRB [9]
Data file 13	Sag018883.gb	GenBank (gb)	Data INRAE, https://doi.org/10.15454/VI7YRB [9]
Data file 14	Sag201008.gb	GenBank (gb)	Data INRAE, https://doi.org/10.15454/VI7YRB [9]
Data file 15	SagNEM316.gb	GenBank (gb)	Data INRAE, https://doi.org/10.15454/VI7YRB [9]
Data file 16	SanC1051.gb	GenBank (gb)	Data INRAE, https://doi.org/10.15454/VI7YRB [9]
Data file 17	ScoC1050.gb	GenBank (gb)	Data INRAE, https://doi.org/10.15454/VI7YRB [9]
Data file 18	ScoC232.gb	GenBank (gb)	Data INRAE, https://doi.org/10.15454/VI7YRB [9]
Data file 19	Sdy12394.gb	GenBank (gb)	Data INRAE, https://doi.org/10.15454/VI7YRB [9]
Data file 20	SdyRE378.gb	GenBank (gb)	Data INRAE, https://doi.org/10.15454/VI7YRB [9]
Data file 21	Seq10565.gb	GenBank (gb)	Data INRAE, https://doi.org/10.15454/VI7YRB [9]
Data file 22	Seq35246.gb	GenBank (gb)	Data INRAE, https://doi.org/10.15454/VI7YRB [9]
Data file 23	Sga2069.gb	GenBank (gb)	Data INRAE, https://doi.org/10.15454/VI7YRB [9]
Data file 24	Sparas15912.gb	GenBank (gb)	Data INRAE, https://doi.org/10.15454/VI7YRB [9]
Data file 25	Spn034183.gb	GenBank (gb)	Data INRAE, https://doi.org/10.15454/VI7YRB [9]
Data file 26	SpnP1031.gb	GenBank (gb)	Data INRAE, https://doi.org/10.15454/VI7YRB [9]
Data file 27	Spy2096.gb	GenBank (gb)	Data INRAE, https://doi.org/10.15454/VI7YRB [9]
Data file 28	SpyHKU.gb	GenBank (gb)	Data INRAE, https://doi.org/10.15454/VI7YRB [9]
Data file 29	SsaFDA259.gb	GenBank (gb)	Data INRAE, https://doi.org/10.15454/VI7YRB [9]
Data file 30	Ssal25975.gb	GenBank (gb)	Data INRAE, https://doi.org/10.15454/VI7YRB [9]
Data file 31	SsalJF.gb	GenBank (gb)	Data INRAE, https://doi.org/10.15454/VI7YRB [9]
Data file 32	SsuBM407.gb	GenBank (gb)	Data INRAE, https://doi.org/10.15454/VI7YRB [9]
Data file 33	SsuNSUI002.gb	GenBank (gb)	Data INRAE, https://doi.org/10.15454/VI7YRB [9]
Data file 34	SsuSC84.gb	GenBank (gb)	Data INRAE, https://doi.org/10.15454/VI7YRB [9]
Data file 35	SsuST1.gb	GenBank (gb)	Data INRAE, https://doi.org/10.15454/VI7YRB [9]
Data file 36	SsuT15.gb	GenBank (gb)	Data INRAE, https://doi.org/10.15454/VI7YRB [9]
Data file 37	SsuZYH33.gb	GenBank (gb)	Data INRAE, https://doi.org/10.15454/VI7YRB [9]
Data file 38	Step12228.gb	GenBank (gb)	Data INRAE, https://doi.org/10.15454/VI7YRB [9]
Data file 39	SthJIM8232.gb	GenBank (gb)	Data INRAE, https://doi.org/10.15454/VI7YRB [9]
Data file 40	StpsHKU10-03.gb	GenBank (gb)	Data INRAE, https://doi.org/10.15454/VI7YRB [9]
Data file 41	Table 1.xlsx	Excel (xlsx)	Data INRAE, https://doi.org/10.15454/VI7YRB [9]

The search for ICEs and IMEs relies on data from the literature and on a semi-automated procedure that was described in [1] and [2]. All annotations were carefully checked and corrected when necessary and almost all of these elements were delineated at the nucleotide level.

ICE expert annotation is based on the identification of genes carried by their conjugation module and encoding three proteins (relaxase, coupling protein and VirB4) needed for their transfer. ICE identification also relies on the search of three types of integrases (tyrosine integrases, serine integrases, and DDE transposases of the IS*Lre2* family). IME characterization was done in a similar fashion as for ICEs except that we searched for genes carried by their mobilization modules encoding a relaxase and eventually a coupling protein.

The co-localization of the genes encoding the signature proteins attests for the presence of an ICE or an IME. Their boundaries were then searched. One relevant information for the ICE/IME delineation is the knowledge of the insertion site targeted by their integrase. To get this information, phylogenetic analyses were done to identify the closest integrase for which the integration site was already identified. ICE/IME boundaries were identified manually by the search for the Direct Repeats (DRs) flanking the elements. When the DRs were missing, too short or too degenerated to be detected, the region containing the element was compared with (i) target regions or genes lacking elements, or (ii) already known and well-delineated elements.

When an expected signature gene was not identified within an element, a thorough examination of all its genes and sequences was done to identify those encoding the missing signature proteins.

## Results

A total of 98 ICEs and 148 IMEs were annotated. We consider as ICEs the elements that were delimited and that encode a relaxase, a coupling protein, a VirB4, and one to three integrases. IMEs are delimited elements that encode one or two relaxases and eventually a coupling protein, both distantly related or unrelated to those of ICEs. We also annotated slightly decayed ICEs and IMEs.

The name of the elements indicates (i) their type (ICE or IME), (ii) the strain of the host bacteria and (iii) the target gene. Elements marked with an asterisk are not integrated in their primary site but in a secondary one. Those marked with “unk” are integrated in a gene of unknown function. The site of insertion of those labeled with ND or NS is non-determined and non-specific, respectively.

In conclusion, the FirmiData dataset constitutes a valuable resource for (i) the microbiologist community interested in mobile genetic elements annotation (ii) the bioinformatics teams developing annotation tools and strategies to detect and annotate mobile genetic elements in bacterial genomes.

## Limitations

- Isolate genes encoding a signature protein or too decayed ICEs or IMEs were not taken into consideration.
- Elements encoding an integrase but devoid of relaxase were not annotated.

## Data Availability

The data described in this Data note can be freely and openly accessed on Data INRAE (https://doi.org/10.15454/VI7YRB). Please see Table 1 and reference [9] for details and links to the data.

## Abbreviations

ICE: Integrative and conjugative element
IME: Integrative and mobilizable element
DRs: Direct repeats
